# Lineage of origin in rhabdomyosarcoma informs pharmacological response

**DOI:** 10.1101/gad.238733.114

**Published:** 2014-07-15

**Authors:** Jinu Abraham, Yaiza Nuñez-Álvarez, Simone Hettmer, Elvira Carrió, Hung-I Harry Chen, Koichi Nishijo, Elaine T. Huang, Suresh I. Prajapati, Robert L. Walker, Sean Davis, Jennifer Rebeles, Hunter Wiebush, Amanda T. McCleish, Sheila T. Hampton, Christopher R.R. Bjornson, Andrew S. Brack, Amy J. Wagers, Thomas A. Rando, Mario R. Capecchi, Frank C. Marini, Benjamin R. Ehler, Lee Ann Zarzabal, Martin W. Goros, Joel E. Michalek, Paul S. Meltzer, David M. Langenau, Robin D. LeGallo, Atiya Mansoor, Yidong Chen, Mònica Suelves, Brian P. Rubin, Charles Keller

**Affiliations:** 1 Pediatric Cancer Biology Program, Papé Family Pediatric Research Institute, Department of Pediatrics, Oregon Health and Science University, Portland, Oregon 97239, USA;; 2 Institute of Predictive and Personalized Medicine of Cancer (IMPPC), Germans Trias i Pujol Health Sciences Research Institute (IGTP), 08916 Badalona, Barcelona, Spain;; 3 Howard Hughes Medical Institute, Department of Stem Cell and Regenerative Biology, Harvard University, Harvard Stem Cell Institute, Joslin Diabetes Center, Cambridge, Massachusetts 02138, USA;; 4 Department of Pediatric Oncology, Dana Farber Cancer Institute, Division of Pediatric Hematology/Oncology, Children’s Hospital, Boston, Massachusetts 02115, USA;; 5 Greehey Children’s Cancer Research Institute, University of Texas Health Science Center, San Antonio, Texas 78229, USA;; 6 Oncogenomics Section, Pediatric Oncology Branch, Advanced Technology Center, National Cancer Institute, Gaithersburg, Maryland 20877, USA;; 7 Department of Neurology, Department of Neurological Sciences, Glenn Laboratories for the Biology of Aging, Stanford University, Palo Alto, California 94304, USA;; 8 Department of Human Genetics, University of Utah, Salt Lake City, Utah 84112, USA;; 9 Institute for Regenerative Medicine, Wake Forest School of Medicine, Winston-Salem, North Carolina 27157, USA;; 10 Department of Epidemiology and Biostatistics, University of Texas Health Science Center, San Antonio, Texas 78229, USA;; 11 Department of Pathology, Center for Cancer Research, Massachusetts General Hospital, Charlestown, Massachusetts 02129, USA;; 12 Department of Pathology, University of Virginia, Charlottesville, Virginia 22903, USA;; 13 Department of Pathology, Oregon Health and Science University, Portland, Oregon 97239, USA;; 14 Department of Anatomic Pathology, Department of Molecular Genetics, Taussig Cancer Center, Lerner Research Institute, Cleveland Clinic Foundation, Cleveland, Ohio 44195, USA

**Keywords:** alveolar rhabdomyosarcoma, Pax3:Foxo1, sarcoma, satellite cell, myoblast, histone

## Abstract

Lineage or cell of origin of cancers is often unknown and thus is not a consideration in therapeutic approaches. Alveolar rhabdomyosarcoma (aRMS) is an aggressive childhood cancer for which the cell of origin remains debated. We used conditional genetic mouse models of aRMS to activate the pathognomonic *Pax3:Foxo1* fusion oncogene and inactivate *p53* in several stages of prenatal and postnatal muscle development. We reveal that lineage of origin significantly influences tumor histomorphology and sensitivity to targeted therapeutics. Furthermore, we uncovered differential transcriptional regulation of the *Pax3:Foxo1* locus by tumor lineage of origin, which led us to identify the histone deacetylase inhibitor entinostat as a pharmacological agent for the potential conversion of Pax3:Foxo1-positive aRMS to a state akin to fusion-negative RMS through direct transcriptional suppression of *Pax3:Foxo1*.

## Abstract (precis)

The cell of origin remains debated for the aggressive childhood cancer alveolar rhabdomyosarcoma (aRMS). Abraham et al. used conditional mouse models of aRMS to activate the Pax3:Foxo1 fusion oncogene and inactivate p53 in several lineages of early development. The results reveal that the tumor cell of origin significantly influences tumor sensitivity to targeted therapies. Furthermore, the transcriptional regulation of the Pax3:Foxo1a locus varies by lineage of origin. These discoveries led to the identification of the histone deacetylase inhibitor entinostat as a potential agent for pharmacological intervention.

Rhabdomyosarcoma is an aggressive solid tumor for which the cell lineage of origin of alveolar rhabdomyosarcoma (aRMS) remains debated (Tiffin et al. 2003; Charytonowicz et al. 2009; Hettmer and Wagers 2010; Hettmer et al. 2011). Whereas the adult pleomorphic subtype is felt to originate from muscle stem cells (satellite cells) (Hettmer et al. 2011; Blum et al. 2013), the embryonal subtype has been attributed to arise more often from myoblasts than upstream myogenic progenitors (i.e., satellite cells), the latter of which are more prone to transform into undifferentiated pleomorphic sarcomas (UPSs) having relatively little myodifferentiation potential (Rubin et al. 2011). Even more intriguing are possible nonmyogenic cells of origin of embryonal rhabdomyosarcoma (eRMS) from the adipogenic lineage (Hatley et al. 2012; Kikuchi and Keller 2012). For the alveolar subtype that is often incurable when metastatic (Malempati and Hawkins 2012; Hawkins et al. 2013), we previously suggested Myf6-expressing (differentiating) cells of the myogenic lineage as the cell of origin (Keller et al. 2004a,b). These studies had used conditional mouse models triggering in the *Myf6Cre* lineage the pathognomonic *Pax3:Foxo1* chimeric oncogene, which is commonly found in the human disease as a result of a t(2;13) translocation (Arndt and Crist 1999). Nevertheless, we and others have reconsidered whether mesenchymal stem cells or satellite cells could be an alternate cell of origin (Ren et al. 2008; Charytonowicz et al. 2009; Hettmer and Wagers 2010), and thus we performed the studies described here.

An interesting aspect of the search for cell of origin is that we uncovered a differential susceptibility of the *Pax3:Foxo1* locus to be transcribed based on the cell lineage originally transformed. This finding may have translational significance in that the related *Pax7* locus in myogenic progenitors is a classic example of a bivalent epigenetic locus (Mozzetta et al. 2011), with different transcriptional activation states depending on degree of myodifferentiation, and thus is potentially amenable to pharmacological intervention. Transcription factors have not typically been considered approachable therapeutic targets, but if transcription of *Pax3:Foxo1* itself could be inhibited, then the implicit conversion of fusion-positive aRMS to fusion-negative RMS would have great appeal, given the substantial difference in outcomes between these two clinical groups in retrospective studies (Sorensen et al. 2002; Missiaglia et al. 2012).

## Results

### The p53 pathway is frequently aberrant in aRMS

We and others have commonly used *p53* inactivation in mouse models of aRMS (Keller et al. 2004a), yet the clinical relevance of *p53* deregulation at the genetic and/or functional pathway levels is debated (Takahashi et al. 2004; Ognjanovic et al. 2012). To address this issue, we used metagene analysis to test whether *p53* functional inactivation was a clinically relevant cooperative initiating mutation in aRMS (Supplemental Fig. S1A). Using our previously described metagene analysis and S-score method (Rubin et al. 2011), we analyzed a global gene expression data set (Supplemental Tables 1, 2) of 62 PAX3:FOXO1^+^ and 24 PAX7:FOXO1^+^ human aRMS tumors for aberrant signaling of the rhabdomyosarcoma-associated p53 pathway. We found that 85% of PAX3:FOXO1^+^ tumors exhibited a gene expression signature consistent with the “p53 off” state. Similarly, 75% of PAX7:FOXO1 tumors also had a p53 off state. Thus, the p53 off state was a common signature in human samples.

### Pax3:Foxo1 leads to forms of sarcoma for satellite cells different from any other myogenic lineage

**Figure 1.**
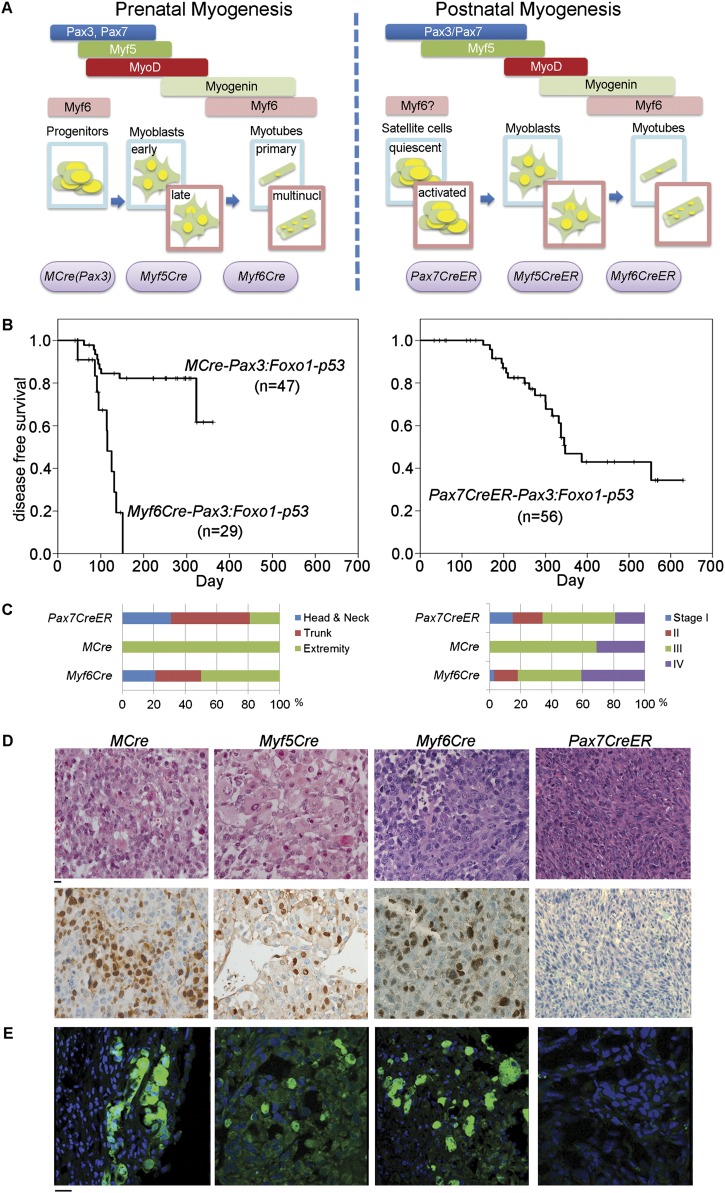
Characteristics of tumors from *Pax7CreER, Myf5Cre*, *Myf6Cre*, and *MCre* mice. (*A*) Representation of prenatal and postnatal myogenesis in the context of Cre drivers used to trigger tumors in this study. For *Pax7CreER*, *Myf5CreER*, and *Myf6CreER* mice, tamoxifen was administered at P30 (see the Materials and Methods). (multinucl.) Multinucleated. (*B*) *Pax7CreER* mice and *MCre* mice showed prolonged survival and lower tumor incidence compared with *Myf6Cre* mice (tumor incidences were *MCre*, 40%; *Pax7CreER*, 38%; and *Myf6Cre*, 100% at 1 yr after birth). *Myf5Cre* mice were embryonic-lethal (see the text). All mice also harbor homozygous *Pax3*^P3Fm/P3Fm^ and *p53*^F2-10/F2-10^ alleles. (*C*) Location and disease stages in each strain. *Pax7CreER* tumors were more frequently observed in head/neck and genital areas, whereas *MCre* mice developed tumors exclusively in the extremities. The highest frequency of stage IV metastasis (40%) was seen for *Myf6Cre* mice. The IRSG staging system was scaled to mice such that a 4-mm-diameter mouse tumor was equated with a 5-cm-diameter human tumor (Nishijo et al. 2009a). (*D*, *top* row) Histological analysis of tumors. *Myf5Cre*, *Myf6Cre*, and *MCre* tumors were small round cells (aRMS), whereas most *Pax7CreER* tumors showed spindle cell morphology (pleomorphic RMS/pleomorphic spindle cell sarcoma). (*Bottom* row) Immunohistochemistry for Myogenin. (*E*) eYFP protein expression representing the transcriptional activation status of *Pax3:Foxo1* fusion gene was at the lower limit of detection in *Pax7-CreER* tumors. Bars, 10 µM.

Having established a prominent role for p53 pathway inactivation and Pax3:Foxo1 activation as driver events in the genesis of aRMS, we generated mouse models (Keller et al. 2004a) to mimic these initiating driver events through targeting to specific muscle cell types in fetal and postnatal development (Fig. 1A). Tumors occurred in embryonic muscle lineages (*MCre* for hypaxial *Pax3*) and embryonic and fetal muscle lineages (*Myf6Cre* for *Myf6*) as well as the postnatal satellite cell lineage (*Pax7CreER*) (Fig. 1B,C). For the latter, tamoxifen was administered at 30 d of age (adolescence). The *Myf5Cre* lineage was embryonic-lethal (usually resulting in exencephaly) (data not shown) in all litters except for one animal (U24014) that later went on to develop a tumor of the lower extremity at 74 d of age. The most susceptible lineages were fetal myogenic progenitors and postnatal satellite cells but not Myf5- or Myf6-expressing postnatal committed myogenic progenitors: Of 18 *Myf5CreER*,*Pax3:Foxo1*,*p53* mice given tamoxifen at 30 d of life (P30 [postnatal day 30]) and observed 43–510 d (median, 395), only one developed at age 401 d a tumor that was diagnosed as aRMS solid variant of the cranial muscle (Supplemental Fig. S1B–D). Similarly, from eight *Myf6CreER*, *Pax3:Foxo1*, *p53* mice given tamoxifen at P30 and observed 232–505 d (median, 396), no mice developed tumors. Postnatal lineage tracing of the postnatal *Myf6CreER* lineage showed marking of myofibers as well as Pax7^+^ sublaminar satellite cells (Supplemental Fig. S2), a postnatal result complementing a report that embryonic *Myf6Cre* might prime Pax7^+^ satellite cells (Sambasivan et al. 2013). However, despite a recent report showing that *Myf6Cre* is active in early embryonic muscle progenitors that can become satellite cells (Sambasivan et al. 2013), other evidence for the *Myf6Cre* fetal myoblast population being a more common cell of origin for Pax3:Foxo1^+^ aRMS than a postnatal satellite cell includes (1) the absence of tumors from *Myf6CreER*, *Pax3:Foxo1*, *p53* mice in which Myf6^+^ satellite cells can activate Pax3:Foxo1, (2) the absence of aRMS tumors from *Pax7CreER*, *Pax3:Foxo1*, *p53* mice in which the Pax7^+^ satellite cell population at large can activate Pax3:Foxo1, and (3) the absence of aRMS tumors from *Myf5CreER*, *Pax3:Foxo1*, *p53* mice whose nonquiescent satellite cells can activate Pax3:Foxo1 (Biressi et al. 2013).

To address the reported possibility that nonmyogenic, marrow-derived cells might give rise to aRMS—at least when Pax3:Foxo1 is driven by a nonnative promoter (Ren et al. 2008)—we first examined cells of the bone marrow compartment for the presence of cells derived from a *Myf6Cre* or *Pax3CreKI* (*Pax3*) (Engleka et al. 2005) ontogeny using eYFP (Keller et al. 2004a) and RFP (Vintersten et al. 2004) reporters, respectively. This lineage tracing was done without introducing *Pax3:Foxo1* or *p53* mutations. Neither the *Pax3* nor the *Myf6* lineage was represented in the bone marrow compartment in >0.4% of mesenchymal or hematopoietic cells (Supplemental Fig. S1E,F), and thus this line of experimentation was discontinued.

**Figure 2.**
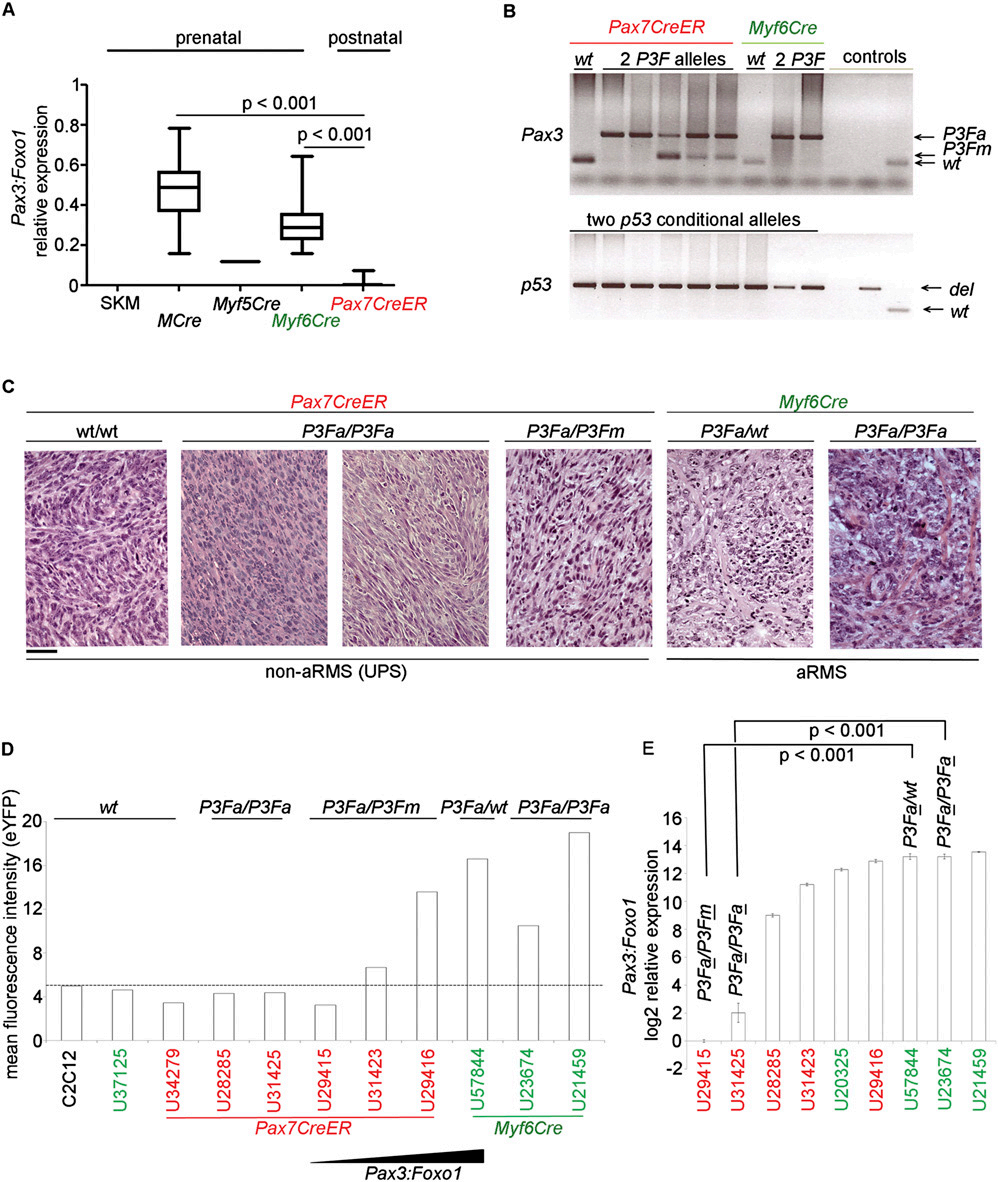
Recombination status of *Pax3* allele differs by cell of origin. (*A*) Relative expression of *Pax3:Foxo1* in mouse tumor tissue. (*B*) Genotyping for *Pax3:Foxo1* and *p53* recombination by cell of origin (Cre driver). Satellite cell-derived (*Pax7CreER*) primary tumor cell cultures showed reduced efficiency in the recombination of *Pax3:Foxo1* knock-in allele locus compared with myoblast-derived primary tumor cell cultures (*Myf6Cre*). However, *p53* locus conditional alleles were uniformly inactivated in all tumors irrespective of Cre driver. (*C*) Representative H&E staining of tumors with Cre driver and *Pax3* locus genotypes as noted. All tumors had homozygous conditional *p53* deletion. *Myf6Cre* lineage tumors with heterozygous or homozygous activation of *Pax3:Foxo1* showed small round cell histology consistent with aRMS. In contrast, *Pax7CreER* tumors with heterozygous or homozygous activation of *Pax3:Foxo1* exhibited pleomorphic spindle cell appearances, similar to tumors with only *p53* inactivation—neither of which appearance is consistent with aRMS. (*D*) Flow cytometry of eYFP for *Pax7CreER*- and *Myf6Cre*-derived primary tumor cell cultures. The mean florescence intensity was lower in *Pax7CreER* rhabdomyosarcoma cells, although select tumors of the *Pax7CreER* lineage (e.g., U31423 and U29416) more resembled *Myf6Cre* in eYFP expression (see the Results). (*E*) Quantitative RT–PCR (qRT–PCR) studies showing higher levels of *Pax3:Foxo1* mRNA levels in *Myf6Cre*-derived primary tumor cell cultures (U23674 and U57844) compared with *Pax7CreER*-derived primary tumor cell cultures (U29415, U31425, and U31423). Statistically significant difference in *Pax3:Foxo1* expression was observed between *Myf6Cre*- and *Pax7CreER*-derived primary tumor cell cultures with one activated *Pax3:Foxo1(P3Fa)* allele (U57844 and U29415) versus primary tumor cultures with two activated *Pax3:Foxo1* alleles (U23674 and U31425).

At the histological level, the embryonic *MCre* (hypaxial *Pax3Cre*) and fetal *Myf6Cre* lineages led to aRMS histology (Fig. 1D). The single animal that survived past birth for the *Myf5Cre* lineage (U24014) also developed aRMS with numerous rhabdoid-type rhabdomyoblasts and diffuse anaplasia (Fig. 1D). In contrast, the *Pax7CreER* postnatal satellite cell lineage led to tumors diagnosed as pleomorphic RMS/pleomorphic spindle cell sarcoma by histological and immunohistochemical criteria (Fig. 1D). Surprisingly, the eYFP marker of *Pax3:Foxo1* was expressed only weakly in *Pax7CreER*-derived tumors compared with the other tumors (Fig. 1E). Correspondingly, *Pax3:Foxo1* expression by RT–PCR was variable and generally low in *Pax7CreER* tumors compared with mice bearing the *Pax3:Foxo1* conditional alleles and other Cre drivers (Fig. 2A).

To begin exploring the potential causes of the low *Pax3:Foxo1* expression from *Pax7CreER,Pax3,p53* tumors, we evaluated the Cre/LoxP recombination efficiency at the *Pax3* locus. By genotyping tumors, *Pax3* locus recombination frequency was found to be lower in tumors of *Pax7CreER-*expressing satellite cell origin than *Myf6Cre*-expressing prenatal (presumably myoblast) origin. Generally *Pax7CreER* tumors had only one *Pax3* conditional knock-in allele, *P3Fm*, converted to the active Pax3:Foxo1 form, *P3Fa* (Fig. 2B; see the Discussion). In contrast, recombination for the *p53* locus by *Pax7CreER* in tumors was uniformly complete for both *p53* alleles. Furthermore, the lower *Pax3* recombination frequency alone would not account for the difference in histology because *Pax3* (*P3Fa/WT*) tumors from the *Myf6Cre* lineage presented with an aRMS histology, not a pleomorphic histology (Fig. 2C; Supplemental Table 3). By quantitative analysis, the mean florescence intensity of eYFP in primary tumor cell cultures derived from *Pax7CreER,Pax3(P3Fm/P3Fa)* tumors was rarely above the level of C2C12-negative control cells except in rare instances (Fig. 2D; see the Discussion). On the other hand, mean fluorescence intensity of eYFP in primary cell cultures derived from a *Myf6Cre*,*Pax3(P3Fa/P3Fa)* tumor was twofold to threefold higher than control C2C12 cells (Fig. 2D). RT–PCR studies also showed higher levels of *Pax3:Foxo1* mRNA levels in murine primary tumor cell cultures derived from *Myf6Cre*,*Pax3(P3Fa/P3Fa)* tumors (Fig. 2E, green) compared with primary tumor cell cultures from *Pax7CreER,Pax3(P3Fa/P3Fa)* tumors (U31425 and U28285) (Fig. 2E). Altogether, these results suggest that inherently less transcription occurs from the *Pax3* locus in *Pax7CreER*-derived tumors and decrease the probability that aRMS arises from Pax7^+^ postnatal satellite cells.

### Tumors arising from non-*Pax7CreER* cell of origin authentically recapitulate human aRMS

**Figure 3.**
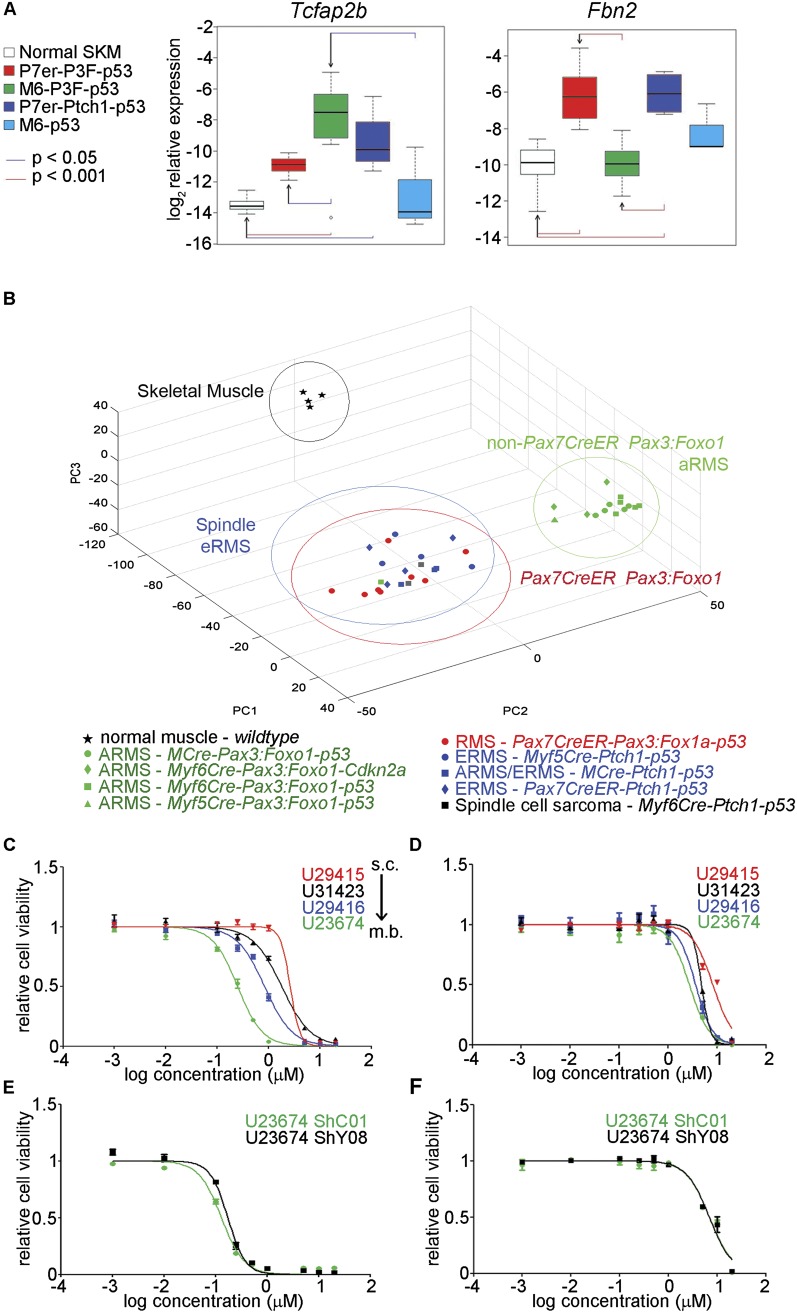
*Pax3:Foxo1* and aRMS-specific marker expression in *Pax3:Foxo1* tumors. (*A*) For tumors bearing conditional *Pax3:Foxo1* alleles, expression of aRMS-specific classifier *Tcfap2b* was significantly increased in *Myf6Cre*-derived tumors (*M6-P3F-p53*) and comparatively lower in *Pax7CreER*-derived tumors (*P7er-P3F-p53*); conversely, expression of eRMS-specific marker *Fibrillin2* (*Fbn2*) was increased in *Pax7CreER*-derived, *Pax3:Foxo1*-bearing tumors and comparable with the level of *Fbn2* from eRMS derived from satellite cells (*P7er-Ptch1-p53*) or myoblasts (*M6-p53*). (*B*) PCA showing the non-satellite cell-derived, *Pax3:Foxo1*-bearing tumors as an outgroup and *Pax7CreER*-derived, *Pax3:Foxo1*-bearing tumors grouping with spindle cell sarcoma/eRMS tumors bearing *Ptch1* and *p53* mutations. (*C*,*D*) Cell viability assay showing differential drug response of primary tumor cell cultures along the continuum of satellite cell-derived (*Pax7CreER*) origin versus myoblast-derived (*Myf6Cre*). (*C*) For the Cdk-2, Cdk-7, and Cdk-9 inhibitor SNS-032, the IC50 values were 0.24 µM for U23674, 0.83 µM for U29415, 1.79 µM for U31423, and 2.61 µM for U29415. (*D*) For the Cdk-4,-6 inhibitor PD0332991, the IC50 values were 2.69 µM for U23674, 3.61 µM for U29415, 4.77 µM for U31423, and 8.15 µM for U29415. (*E*,*F*) Cell viability assay showing no difference in the response of U23674 ShY08 cells (*Pax3:Foxo1* stable knockdown cells) to SNS-032 (*E*) and PD0332991 (*F*) compared with the control knockdown cells, U23674 ShC01. For SNS-032, the IC50 values were 0.1305 µM for ShC01 cells and 0.1730 µM for ShY08 cells. In the case of PD0332991, the IC50 values were 6.90 µM for ShC01 cells and 6.88 µM for ShY08 cells. (s.c.) Satellite cell origin; (m.b.) myoblast origin.

To further evaluate the lineages that might give rise to aRMS, we compared expression of validated markers of Pax:Foxo1^+^ aRMS and eRMS (Wachtel et al. 2006). *Myf6Cre* myoblast-derived mouse tumors expressed significantly higher levels of the Pax3:Foxo1-specific aRMS marker *Tcfap2b*, whereas *Pax7CreER* satellite cell-derived tumors expressed reciprocally higher levels of the eRMS-specific marker *Fbn2* (Fig. 3A). We then turned to principal component analysis (PCA) to compare Pax3:Foxo1-bearing tumors with those in the eRMS–UPS spectrum (Rubin et al. 2011). Results showed that *Pax7CreER* satellite cell-derived tumors were highly similar to eRMS/UPS tumor models that we previously reported (Fig. 3B; Rubin et al. 2011). In contrast, all Pax3:Foxo1-bearing tumors histologically consistent with aRMS from the *MCre* (*Pax3*), *Myf5Cre*, and *Myf6Cre* lineages formed an independent outgroup (Fig. 3B). Thus, the *Pax7CreER* postnatal satellite cell lineage did not appear to be poised for giving rise to aRMS when the *Pax3:Foxo1* oncogene at the *Pax3* locus is triggered; instead, cells of *Pax7CreER* satellite cell lineage had a tendency to give a spindle cell-like tumor phenotype. We next hypothesized that lineage-specific drug sensitivity could be best interrogated by an ontogeny series that would include *Pax7CreER* tumors for which Cre-mediated recombination or transformation occurred after the satellite cell state. From Figure 3B, we expected an intermediate state to be rare, but from the *Pax3:Foxo1* data in Figure 2E (i.e., samples U28285, U31423, and U29416), we expected at least some instances to exist. To identify a potential ontogeny series, we first sought to find model primary cell cultures best representing Pax3:Foxo1-harboring satellite cell (*Pax7CreER* lineage)-derived tumors versus Pax3:Foxo1-harboring myoblast (*Myf6Cre* lineage)-derived tumors. Comparison of gene expression for these two tumor types is given in Supplemental Table 4. The *Pax7CreER* lineage was associated with expression of *Cav1*, *Dcn*, and *Cxcl12*, whereas the *Myf6Cre* lineage was associated with expression of *Sct*, *Stc1*, and *Ryr3* (Supplemental Table 4). We choose five primary cell cultures—i.e., U29415, U31425, U28285, U31423, and U29416—of *Pax7CreER* lineage and U20325, U23674, and U21459 of *Myf6Cre* lineage to test the expression of *Cav1*, *Dcn*, *Cxcl12*, *Sct*, *Stc1*, and *Ryr3* by RT–PCR. These studies showed an interesting gradient pattern in which the expression of markers associated with *Pax7CreER* lineage was highest in U29415 cells followed by U31425 or U28285 and lower expression in *Myf6Cre* lineage primary cell cultures U20325, U23674, and U21459. Conversely, the expression of markers associated with *Myf6Cre* lineage showed a reverse gradient pattern where the highest expression was observed in U21459/U23674, followed by U29416 or U20325, U31423, U28285, U31425, and U29415 cells (Supplemental Fig. S3A). These results suggested that U31425, U28285, U31423, and U29416 represent the “intermediate” myodifferentiation and transformation between U29415 (*Pax7CreER*) and U23674/U21459 (*Myf6Cre*) (Supplemental Fig. S3A). Having this ontogeny series in hand, we sought to test the therapeutic significance of the difference in cell of origin by subjecting the ontogeny series of primary cell cultures to cell cycle-specific inhibitors that we reasoned from the literature (Jahn et al. 1994; De Falco and De Luca 2006) may have differential sensitivity to cells of increasing myodifferentiation. The four cell cultures did indeed have differential sensitivities to the Cdk-2, Cdk-8, and Cdk-9 inhibitor SNS-032 and the Cdk-4/6 inhibitor PD0332991 (Fig. 3C,D). The primary tumor cell culture U23674 (*Myf6Cre* myoblast origin) was found to be the most sensitive to SNS-032 and PD0332991 compared with the other three primary tumor cell cultures (*Pax7CreER* satellite cell lineage), suggesting that the cell of origin of the sarcoma might play a role in deciding the treatment strategy for patients. Similar results were observed for the FGFR1–4 inhibitor, LY287445, and Wee1 inhibitor, MK-1775, whereby *Myf6Cre* lineage-derived U23674 was found to be the most sensitive to these two drugs compared with intermediate ontogeny cultures U29416 and U31423 and *Pax7CreER* satellite cell lineage-derived U29415 (Supplemental Fig. S3C,D). Interestingly, when *Pax3:Foxo1* was stably knocked down in U23674 cells (U23674 ShY08), there was no difference in their sensitivity to either SNS-032 (Fig. 3E) or PD0332991 (Fig. 3F) compared with the control cells (U23674 ShC01), further supporting that differential drug sensitivity was due to lineage of origin (i.e., epigenetic memory) and not *Pax3:Foxo1* expression level.

### The *Pax7* and *Pax3:Foxo1* loci are epigenetically regulated (differential effects of epigenetic modifiers on *Pax3:Foxo1* and *Pax7* expression depending on the tumor cell of origin)

We next reasoned that if tumor initiation and tumor phenotype is epigenetically determined, then the tumor phenotype can be epigenetically modified by pharmacological agents. The *Pax7* locus in activated satellite cells has a bivalent chromatin domain with the presence of both positive (H3K4me3) and negative (H3K27me3) histone marks (Mozzetta et al. 2011), and we hypothesized that the *Pax3* locus may be similar. To gain insight into the epigenetic regulation of the *Pax3:Foxo1* locus, we examined active (H3K4me3 and H3K9Ac) and repressive (H3K27me3 and H3K9me3) histone modification marks by chromatin immunoprecipitation (ChIP) in tumor cells representing both *Pax7CreER* (U29415 and U31425) and *Myf6Cre* (U23674 and U21459) lineages of origin. For this study, we analyzed two genomic regions upstream (−193: −285) of and downstream (+593: +462) from the transcriptional start site (TSS) of the *Pax3* locus. At the level of histone marks, we observed a different chromatin state of the *Pax3:Foxo1* locus in primary tumor cell cultures with different lineages of origin. As shown in Supplemental Figure S4, higher levels of both H3K4me3 and H3K9Ac active chromatin marks are observed in U23674 and U21459 cells compared with U29415 and U31425 tumor cells, correlating with high versus low *Pax3:Foxo1* expression, respectively. Also high levels of repressive histone marks H3K27me3 and H3K9me3 were observed in U29415 and U31425 cells compared with U23674 and U21459 cells. Altogether, these data suggest that the observed differences in *Pax3:Foxo1* mRNA levels could be explained by a different chromatin state in these loci depending on the tumor lineage of origin. From the RT–PCR studies described above, U29415 was selected as a representative *Pax7CreER* lineage tumor culture, and U23674 was selected as a representative *Myf6Cre* lineage tumor culture.

**Figure 4.**
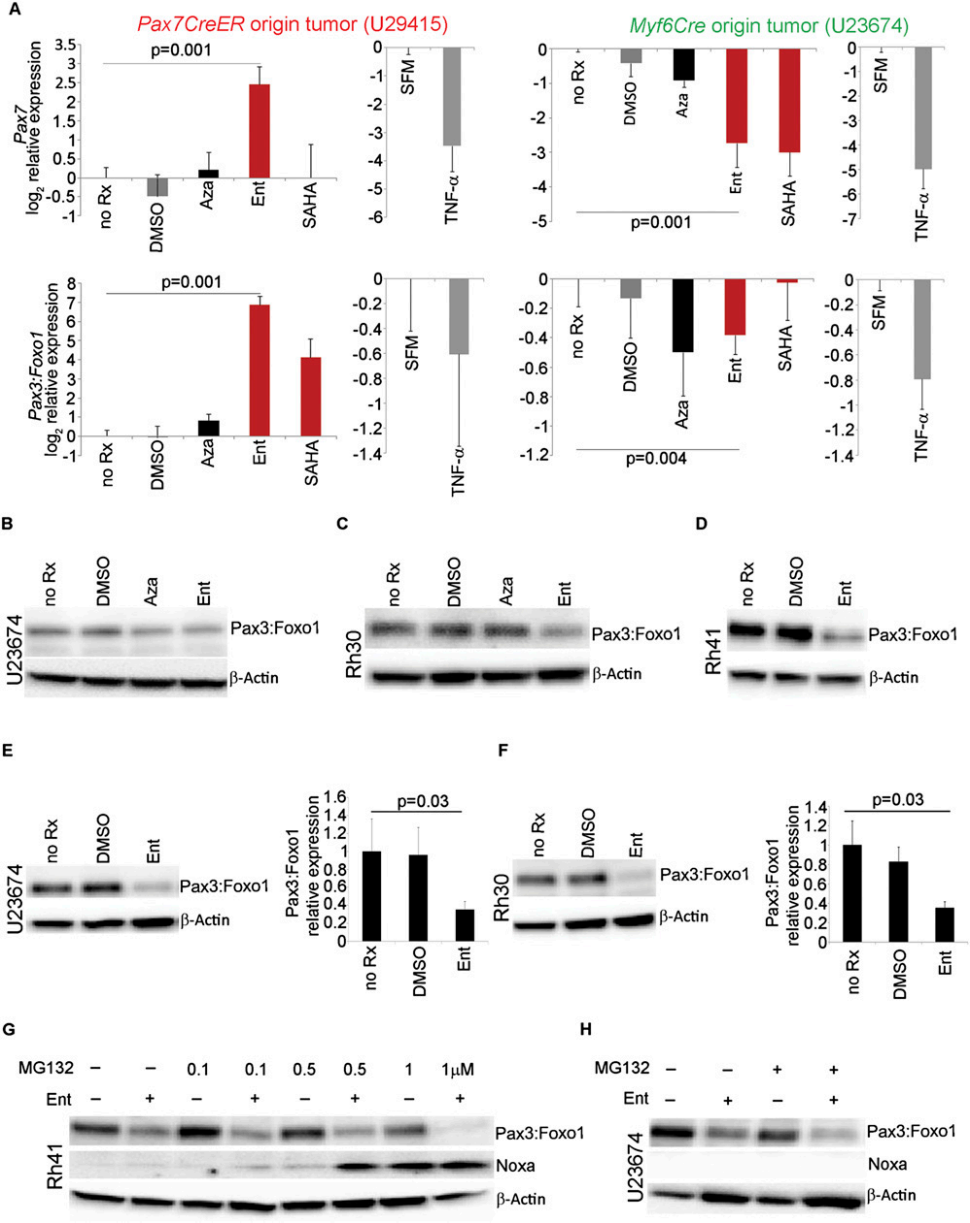
Pharmacologically modified epigenetic regulation of Pax3:Foxo1. (*A*) RT–PCR assays showing relative expression of *Pax3:Foxo1* and *Pax7* in representative satellite cell-derived U29415 and myoblast-derived U23674 murine primary tumor cell cultures upon treatment with DMSO, 5-Aza-2′deoxycytidine (Aza), SAHA, entinostat (Ent), serum-free medium (SFM), and the cytokine TNF-α (10 ng/mL in serum-free medium) for 24 h. The U29415 primary tumor cell culture (satellite cell origin) showed an increase in *Pax7* and *Pax3:Foxo1* expression upon treatment with 5-Aza-2′deoxycytidine, SAHA, and entinostat for 24 h, whereas the U23674 primary tumor cell culture (myoblast origin) showed a decrease in the mRNA levels of *Pax7* and *Pax3:Foxo1* upon treatment. However, treatment with TNF-α showed a reduction in the expression of *Pax7* and *Pax3:Foxo1* in both U29415 and U23674 cells. (*B*) Immunoblot showing reduction in Pax3:Foxo1 levels upon treatment with 2 µM entinostat (Ent) and also with 3 µM 5-Aza-2′deoxycytidine (Aza) for 24 h in U23674 cells. (*C*) Reduction in Pax3:Foxo1 level upon treatment with 2 µM entinostat (Ent) for 24 h in Rh30 cells. (*D*) Western blot showing reduction in Pax3:Foxo1 levels upon treatment with 2 µM entinostat (Ent) for 24 h in Rh41 cells. (*E*) Immunoblot showing significant reduction in Pax3:Foxo1 levels upon treatment with 2 µM entinostat (Ent) for 72 h in U23674 cells (*left* panel) and the graphical representation (*right* panel). (*F*) Immunoblot showing a significant reduction in Pax3:Foxo1 level upon treatment with 2 µM entinostat (Ent) for 72 h in Rh30 cells (*left* panel) and the graphical representation for this blot (*right* panel). (*G*) Western blots showing no increase in Pax3:Foxo1 levels in Rh41 cells when they were treated with a combination of the proteasome inhibitor MG132 (0.1 µM, 0.5 µM, and 1 µM) and 2 µM entinostat for 24 h compared with cells treated with entinostat alone. Expression of Noxa was observed when cells were treated with a combination of MG132 and entinostat in Rh41 cells. (*H*) Western blot showing no increase in Pax3:Foxo1 levels in U23674 cells even when these cells were treated with a combination of 200 nM MG132 and 2 µM entinostat for 72 h compared with cells treated with 2 µM entinostat alone. Noxa could not be detected even after treating the cells with a combination of MG132 and entinostat for 72 h.

To determine whether the *Pax* locus could be pharmacologically modified, we first treated murine primary tumor cell cultures U23674 and U29415 with varying doses of 5-Aza 2′deoxycytidine (DNA methyltransferase inhibitor) or SAHA and entinostat (histone deacetylase [HDAC] inhibitors) to determine a sublethal dose at which cells could be treated to study the effects of these drugs on expression of *Pax3:Foxo1* and *Pax7* (Supplemental Fig. S5). Cells were treated with drugs for 72 h, after which an MTS assay was performed to assess viability. Based on these studies, U23674 cells were treated with 3 µM 5-Aza-2′deoxycytidine, 2 µM entinostat, or 0.75 µM SAHA for 24 h, after which RNA was extracted, and the expression of *Pax3:Foxo1* and *Pax7* was examined by RT–PCR. For U23674 tumor cells of the *Myf6Cre* myoblast lineage, a decrease in both *Pax7* and *Pax3:Foxo1* mRNA levels upon treatment with all of the drugs was observed, but the effect was more pronounced for HDAC inhibitors (Fig. 4A). Interestingly, U29415 tumor cells of *Pax7CreER* satellite cell lineage showed a paradoxical increase in mRNA levels of *Pax7* and *Pax3:Foxo1* when treated with 20 µM 5-Aza-2′deoxycytidine, 15 µM entinostat, and 5 µM SAHA (Fig. 4A). As expected, the rare tumor cultures downstream from U29415 behaved similarly to the *Myf6Cre* tumor cultures (Supplemental Fig. S6). We next determined epigenetic marks of *Pax3:Foxo1* by performing ChIP and examining the active histone marks H3K4me3 and H3K9Ac along with the repressive marks H3K27me3 and H3K9me3 previously examined in Supplemental Figure S4. However, examination of these specific chromatin marks did not explain the change in *Pax3:Foxo1* transcript levels, implying that other regions may be the targets of chromatin remodeling for entinostat.

Previous studies in *mdx* mice treated with a TNF-α antibody reported increased levels of *Pax7* mRNA in satellite cells derived from myofibers (Palacios et al. 2010). TNF-α has been shown to activate p38α kinase, which leads to interaction between YY1 and PRC2 (polycomb-repressive complex 2) via threonine phosphorylation of EZH2. These proteins together form a repressive complex at the *Pax7* promoter and thus regulate the expression of Pax7 (Palacios et al. 2010). To investigate whether TNF-α can regulate the expression of *Pax7* in U23674 and U29415 tumor cells, cultures were treated with 10 ng/mL recombinant TNF-α for 24 h. RT–PCR data showed a reduction in mRNA levels of *Pax7* in both U23674 and U29415 cells. TNF-α also reduced *Pax3:Foxo1* levels in both cell cultures (Fig. 4A). We next examined the levels of Pax3:Foxo1 at the protein level. By immunoblotting, concomitant reduction in Pax3:Foxo1 levels in U23674 cells treated with 2 µM entinostat for 24 h was observed (Fig. 4B). This effect was seen across species for human aRMS cell lines Rh30 (24 h of treatment) and Rh41 (24 h of treatment) (Fig. 4C,D). When murine and human tumor cultures were treated with 2 µM entinostat for 72 h, a pronounced reduction in Pax3:Foxo1 protein levels was observed (Fig. 4E,F). These results showed in principle that *Pax3:Foxo1* and *Pax7* transcription and protein levels can be modified pharmacologically. To investigate whether the reduction in Pax3:Foxo1 protein levels upon entinostat treatment is mediated by changes in post-translational stability attributable to direct HDAC–Pax3:Foxo1 interactions, we treated human and murine aRMS cultures (Rh41 for 24 h and U23674 for 72 h) with 2 µM entinostat alone or in combination with MG132 (proteasome inhibitor). No increase in Pax3:Foxo1 protein level was observed when cells were treated with a combination of entinostat and MG132 compared with cells treated with entinostat alone (Fig. 4G,H). This result suggests that the reduction in Pax3:Foxo1 levels observed upon entinostat treatment is mediated at the transcriptional (or post-transcriptional) level and not at the post-translational level. Other studies from our laboratory have shown that expression of *Pax3:Foxo1* is G_2_ cell cycle phase-dependent (Kikuchi et al. 2014). To investigate whether the reduction in Pax3:Foxo1 expression caused by entinostat treatment is cell cycle-dependent, we performed a series of studies suggesting that this was not necessarily the case (Supplemental Fig. S7).

### Entinostat slows tumor growth in vivo

**Figure 5.**
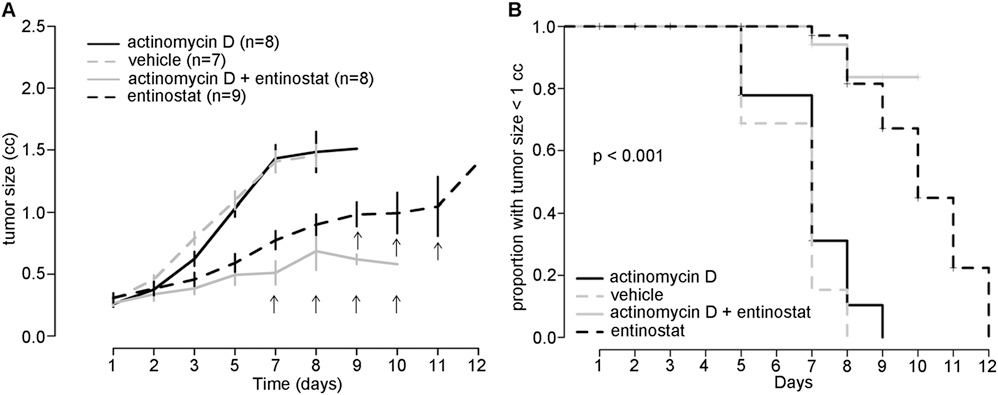
Anti-tumor activity of entinostat in an orthotopic allograft mouse mouse model of rhabdomyosarcoma. (*A*) Tumor-bearing mice were treated with entinostat (dotted black line; *n* = 9; 10 mg/kg daily by intraperitoneal route), actinomycin D (black line; *n* = 8; 0.25 mg/kg on day 1 of treatment by intraperitoneal route), entinostat + actinomycin D (gray line; *n* = 8), and DMSO (dotted gray line; *n* = 7). The graphs shows mean tumor size ± SE by treatment and time. Tumor size increased monotonically with time after treatment with actinomycin D, DMSO, and entinostat and exhibited a downward trend after day 8 for the treatment group with actinomycin D + entinostat. The black arrows indicate the days on which entinostat- or entinostat + actinomycin D-treated mice were sacrificed for 10%–15% body weight loss. (*B*) Kaplan-Meier survival curves for tumor-bearing mice treated with entinostat daily, actinomycin D on day 1, entinostat + actinomycin D, and vehicle (DMSO), with endpoint measurement being the number of days taken for the tumor to reach 1 cm^3^ in size. A null hypothesis that the treatment-specific survival curves are equal was tested using a log-rank test and concluded that the four curves are significantly different (*P* < 0.001).

We next tested the anti-tumor efficacy of entinostat in an orthotopic allograft model of rhabdomyosarcoma generated by injecting U23674 cells into the cardiotoxin-preinjured gastrocnemius muscle in the right leg of SCID-hairless (SHO-*Prkdc*^*scid*^ *Hr*^*hr*^) mice. Once the tumors reached 0.25 cm^3^, the mice were treated with a daily dose of 10 mg/kg entinostat by intraperitoneal injection, with 0.25 mg/kg actinomycin D by intraperitoneal injection on day 1 of treatment, a combination of entinostat and actinomycin D, or vehicle (DMSO) (Fig. 5A,B). Actinomycin D is one of the commonly used chemotherapeutic agents for treating rhabdomyosarcoma. The tumors in the entinostat cohort grew significantly slower than either vehicle- or actinomycin D-treated mice (*P* = 0.03). Mice treated with a combination of entinostat and actinomycin D had a significantly slower rate of tumor growth, and their tumors took more time to reach 1 cm^3^ in size compared with vehicle-treated or actinomycin D alone-treated mice (*P* < 0.001) (Fig. 5 A,B). However, after 5 d of treatment with entinostat, the mice showed symptoms of drug toxicity (>10% loss of body weight), and hence no treatment was administered on day 6, and the subsequent daily dose of entinostat was reduced to 5 mg/kg.

## Discussion

In our studies, we sought to refine experimentally the range of possibilities for the cell of origin of Pax3:Foxo1^+^ rhabdomyosarcoma. Parenthetically, however, the cell of origin for Pax7:Foxo1^+^ aRMS is in no way addressed in this study. Surprisingly, in our experiments, the fetal myoblast (*Myf6* lineage) appears most susceptible to formation of metastatic aRMS. In contrast, muscle stem cells (satellite cells) appear least poised to give rise to rhabdomyosarcoma—at least from adolescence onward. With regard to tumor initiation, our data suggest that the *Pax3* chromatin structure may be closed (and relatively inaccessible to events like Cre/LoxP DNA recombination) and show that transcription from the *Pax3* locus is inactive in tumor cells of this satellite cell lineage. However, this low transcriptional activity could be reversed by pharmacological epigenetic modifiers. Even more importantly, *Pax3:Foxo1* can be reduced at the mRNA and protein level in myoblast-derived tumors by the same pharmacological epigenetic modifiers. The implication that cell of origin can influence pharmacological response is interesting, if not provocative. We concede, however, that common drug sensitivities are likely also found between tumors of different origins and that while some cell biological properties that may be transmitted from the cell of origin, as many or more may be the result of convergent, facultative phenotypes for tumor cell growth—even if rhabdomyosarcomas arise from different cell lineages.

What is remarkable is that a prenatal (*Myf6Cre*) lineage of origin should, in our comparison, be most capable of forming aRMS (at least in the context of p53 loss of function). Rhabdomyosarcoma is a disease that has a steady incidence throughout childhood (aRMS contrasts dramatically with eRMS, the latter of which peaks with toddler and adolescent growth spurts) (Ognjanovic et al. 2009). Suggestion of Myf6^+^ prenatal or postnatal myogenic cells, possibly a terminally differentiating subpopulation, as an aRMS cell of origin was first reported by our group (Keller et al. 2004a,b) and later complemented by in vitro studies of human postnatal myoblasts transformed by *PAX3:FOXO1* and other cooperative initiating mutations (Naini et al. 2008). Congenital fusion-positive aRMS is rarely reported, and congenital aRMS is generally fusion-negative (Godambe and Rawal 2000; Grundy et al. 2001; Sueters et al. 2005), although the *PAX3:FOXO1* translocation has been detected in rare neonatal instances (Rodriguez-Galindo et al. 2001). What might account for the delay? One explanation might be an accumulation of other genetic changes (mutations), but epigenetic changes during childhood and adolescent growth and development might also have a role to play. In fact, epigenetic abnormalities are very common in human cancers—and, in contrast to genetic mutations, these epigenetic changes are potentially reversible.

Can cells other than those of skeletal muscle lineages give rise to aRMS? We are increasingly intrigued by this possibility, although the question needs to be addressed with prudence. A mesenchymal stem cell origin for aRMS has been suggested (Charytonowicz et al. 2009; Hettmer and Wagers 2010). In support of this possibility, clinical cases of metastatic tumors without primary tumors have been reported for t(1;13) PAX7:FOXO1 aRMS (Lisboa et al. 2008) and t(2;13) PAX3:FOXO1 aRMS (Sandberg et al. 2001; Lisboa et al. 2008). An experimental study by Ren et al. (2008) tested the possibility that constitutively expressed (ectopic) *Pax3:Foxo1* could create aRMS, but this study did not place *Pax3:Foxo1* under the authentic control of the *Pax3* promoter, which, in light of our studies of a very small (0.4%) bone marrow compartment cell population that had ever expressed Pax3, makes the probability of aRMS arising from mesenchymal stem cells very low. However, a nonmarrow mesenchymal stem cell of origin remains possible.

An interesting alternative cell of origin for aRMS is the pericyte or mesangioblast. Vascular mimicry in rhabdomyosarcoma suggests a plasticity that may (or may not) have carried over from an angioblast origin (Sun et al. 2004). An interesting case of primary rhabdomyosarcoma of the pulmonary artery was also recently reported (Suman et al. 2013). Experimentally, Lagha et al. (2009) reported that in the mouse embryonic dermamyotome, the ratio of Pax3 to Foxc2 balances fate between myogenic and vascular cell fates but that Pax3:Foxo1 suppresses *Foxc2*. This latter result suggests that, in the susceptible cell, an angiogenic precursor might be converted to a myogenic state, a result that has been shown experimentally for embryonic aortic mesangioblasts (Messina et al. 2009). The link between vascular precursors and the *Pax3* lineage does not come as a surprise given the known role of Pax3 in the migration of cardiac neural crest during embryogenesis (Conway et al. 1997). In a provocative study, Goupille et al. (2011) have shown that smooth muscle cells of arteries express *Pax3* and are able to contribute to skeletal muscle fiber formation via in vitro coculture conditions and following cell–cell fusion. This plasticity lends itself to the possibility that mesangioblasts or even smooth muscle cells can transform into myogenic phenotype cancers (rhabdomyosarcomas) under select conditions.

Our results raise a potentially important clinical implication by suggesting that *PAX3:FOXO1* levels vary by cell of origin, even adjusting for recombination frequency; thus, *PAX3:FOXO1* RT–PCR alone may be less sensitive than cytogenetics or FISH to detect the PAX3:FOXO1 rearrangements. Could some MFH or UPS cases be in fact a spindle cell variant of aRMS, arising from satellite cells? Pathologists might consider addressing this possibility in cases of pediatric and adolescent undifferentiated sarcomas and consider FISH for PAX3:FOXO1 in atypical cases.

Translationally, our data suggest a mechanism by which fusion-positive aRMS might be epigenetically reprogrammed into fusion-negative RMS using the adult phase II HDAC inhibitor entinostat. Transcription factors were once considered undruggable, but our approach complements other experimental demonstrations for EWS:FLI1 in pediatric Ewing’s sarcoma (Barber-Rotenberg et al. 2012) and MLL-associated pediatric leukemias (Chen et al. 2013). While additional preclinical studies remain to be done, it is exciting to consider that entinostat might reduce *PAX3:FOXO1* levels sufficiently to convert fusion-positive RMS with a poor prognosis to a biological state akin to fusion-negative RMS, which portends a significantly better prognosis even when metastatic (Sorensen et al. 2002; Missiaglia et al. 2012).

## Materials and methods

### Microarray analysis for S scores and PCA

Human microarray data sets were previously published (Wachtel et al. 2004; Lae et al. 2007; Davicioni et al. 2009). Patient demographics are presented in Supplemental Table 1, A and B. Metagene and S-score analyses were conducted as previously described (Rubin et al. 2011). For mouse tumors, gene expression analysis was performed using Illumina’s Mouse Ref 8 Beadchip version 1 (Illumina, Inc.). These data sets have been deposited in Gene Expression Omnibus as accession entry GSE22520 and are also described in Supplemental Table 1C. Rank invariant normalization was performed on the log_2_ transformed expression value. Afterward, we applied PCA to all mouse tissue samples with the 19,070 probes (selected with the criterion of average log_2_ intensity >5.5 and standard deviation >0.1) and then plotted in three-dimensional space for visualization (Fig. 4B). Similar microarray data analysis and the PCA methods were previously described (Rubin et al. 2011). All bioinformatics tasks were performed with MATLAB/Bioinformatics Toolbox (MathWorks, Inc.) unless otherwise noted.

We downloaded the public domain data sets for fusion-negative RMS reported by Davicioni et al. (2006) from https://array.nci.nih.gov/caarray/project/details.actionproject.experiment.publicIdentifier=trich-00099 as well as aRMS rhabdomyosarcoma data sets from previously published reports (Wachtel et al. 2004; Lae et al. 2007). These fusion-negative RMS and aRMS data sets were designated as the test samples, whereas normal skeletal muscle samples reported by Bakay et al. (2002) were used as the control group. We also downloaded signature-specific data sets as described below. All of the studies were performed on an Affymetrix U133A array platform (Affymetrix). Sample IDs used are given in Supplemental Tables 1 and 2. Control samples of each examination are the eRMS samples derived from Rubin et al. (2011). We selected 10 high positive, 10 low positive, and 10 negative control samples for each examination, whereas gene-wise *t*-tests were performed between the 20 positive controls and normal skeletal muscle in order to apply to S score. S score is a subtype scoring method to quantify each sample’s consistency. By using the S score, we can unambiguously identify sample status and the amplitude of pathways or biological processes that gene signatures have depicted. The detailed S-score method has been previously described (Rubin et al. 2011).

We used previously established gene lists (Rubin et al. 2011) to examine whether human fusion-negative RMS and aRMS tumors had evidence of p53 loss of function, Shh gain of function, pRb loss of function, or Ras activation. Gene signatures of p53 loss of function were derived from gene expression data sets in breast cancer (Miller et al. 2005). We also downloaded data sets for medulloblastoma samples known to exhibit a Shh gain-of-function signature (Thompson et al. 2006). We took homolog genes from *Rb1* wild-type and homozygous *Rb1*-deleted fusion-negative mouse sarcomas to be pRb loss-of-function gene signature (Rubin et al. 2011). For Ras activation, we used gene lists for the activated Ras signature of zebrafish eRMS (Langenau et al. 2007). The details of obtaining gene signature of each case have been previously described (Rubin et al. 2011).

### Mice

All animal procedures were conducted in accordance with the Guidelines for the Care and Use of Laboratory Animals and were approved by the Institutional Animal Care and Use Committee (IACUC) at the University of Texas Health Science Center at San Antonio or Oregon Health and Science University. The *Myf6Cre*, *Myf5Cre*, *Pax7Cre*, *MCre*, *Pax7CreER*, *Pax3:Foxo1*, *LUSEAP*, LacZ reporter (*Rosa26*^tm1Sor^), Pax3-Cre (*Pax3*^CreKI^), and conditional *p53* mouse lines and corresponding genotyping protocols have been described previously (Soriano 1999; Marino et al. 2000; Jonkers et al. 2001; Keller et al. 2004a; Brown et al. 2005; Nishijo et al. 2009a,b). *Myf5CreER* have been reported (Biressi et al. 2013), and *Myf6CreER* mice will be reported in detail elsewhere but are also described in Supplemental Figure S2. For *Pax7CreER*, *Myf5CreER*, and *Myf6CreER* mice, tamoxifen was administered at P30 as previously reported (Keller et al. 2004a). Tumor-prone mice were visually inspected every 2 d for tumors because of the fulminant onset in these models. Genotyping protocols for *Pax3:Foxo1* and *p53* were performed as described (Keller et al. 2004a).

### Mouse primary tumor cell cultures

Mouse primary tumor cell cultures were generated from mouse tumors as described previously (Abraham et al. 2011).

### Survival analysis and statistical methods

Kaplan-Meier survival analysis of the mice was performed with the endpoint being the development of tumors (i.e., disease-free survival). Survival plots were created using the survival package in R. Relative expression of *Pax3:Foxo1* values were compared using the Mann-Whitney test (Fig. 2A). The log-rank test (Fig. 3A) was used to determine the statistical significance (*P* < 0.05) using Systat12 software (Systat Software, Inc.). The expression of *Pax7* and *Pax3:Foxo1* compared with corresponding reference groups was done using the Mann-Whitney test with the Hochberg correction for multiple testing (Fig. 4A). Statistical analyses were performed using R 3.0.1 (The R Foundation for Statistical Computing, http://www.r-project.org).

### RNA isolation and quantitative RT–PCR (qRT–PCR)

RNA isolation and RT–PCR from mouse tumors were performed as previously described (Nishijo et al. 2009a; Rubin et al. 2011). Probe sets for mouse samples were *Gapdh*-Mm99999915_g1, *Fbn2*_Mm00515742_m1, and *Tcfap2b*_Mm00493468_m1. Statistical analysis was performed by Kruskal-Wallis test with Tukey’s multiple testing correction. For drug treatment studies, the myoblast-derived primary tumor cell culture U23674 was treated with either 750 nM SAHA, 3 µM 5-Aza-2′deoxycytidine, or 2 µM entinostat, and the satellite cell-derived primary tumor cell culture U29415 was treated with either 5 µM SAHA, 20 µM 5-Aza-2′deoxycytidine, or 15 µM entinostat for 24 h. After drug treatment, total RNA was isolated, and cDNA was synthesized as described above. The expression of *Pax3:Foxo1* and *Pax7* was determined by qRT–PCR using TaqMan primers and probes (mouse *Pax7*-Mm00834032_m1) on a StepOnePlus real-time PCR system from Applied Biosystems.

### Histology*,* immunohistochemistry*,* and immunochemistry

For MyoD, Myogenin, and Desmin immunohistochemistry, staining was performed as previously described (Rubin et al. 2011). Immunofluorescence for GFP has also been described previously (Nishijo et al. 2009a). For immunocytochemistry, murine rhabdomyosarcoma primary tumor cell cultures (U23674) and human rhabdomyosarcoma cell lines (Rh41) were cultured in an eight-well CultureSlide (BD Falcon). The cells were washed with PBS, fixed with 4% paraformaldehyde, permeabilized with 0.25% Triton X-100, and incubated with the antibody overnight at 4°C. The primary antibodies used were mouse anti-Pax3 (R&D Systems), rabbit anti-phospho CDC2-Y15, and rabbit anti-phospho Histone H3 (Cell signaling Technology). After incubation, the cells were washed with PBS and incubated with fluorescein isothiocyanate-conjugated anti-mouse and anti-rabbit IgG (Invitrogen) for 1 h. The cells were then examined by using a Zeiss LSM700 confocal microscope and Zen 2010 imaging software (Carl Zeiss).

### Flow cytometry and cell cycle analysis

eYFP expression in mouse rhabdomyosarcoma cells and bone marrow aspirates was assessed by flow cytometry. Cells were stained with propidium iodide at 20 μg/mL for dead cell elimination, followed by analysis on a FACScan cytometer (Becton Dickinson). For cell cycle analysis, primary mouse tumor cell cultures were harvested, washed with PBS, and fixed in cold 70% ethanol for 20 min. After fixation, the cells were washed with PBS and then incubated with RNase A and 20 µg/mL propidium iodide. The cells were then analyzed by using an Influx flow cytometer (Becton Dickinson) and FlowJo software (Tree star, Inc.).

### Western blotting

Procedures have been described previously (Abraham et al. 2011). The following primary antibodies were used: goat anti-FKHR (catalog no. sc-9808, Santa Cruz Biotechnology) to detect Pax3:Foxo1 and mouse anti-Noxa (catalog no. ab13654, Abcam).

### Drug sensitivity assays

Murine rhabdomyosarcoma primary tumor cell cultures were plated in a 96-well plate at 2500 per well. After 24 h, the cells were incubated with varying concentrations of entinostat (Selleckchem), SAHA (LC Laboratories), and 5-Aza-2′deoxycytidine (Sigma-Aldrich) for 3 d. The cytotoxic effect of the drugs was then assessed by using CellTiter 96 AQueous One cell proliferation assay (MTS) and a BioTek Synergy 2 plate reader (BioTek).

### Stable knockdown of *Pax3:Foxo1*

Murine primary tumor cell cultures with stable knockdown of *Pax3:Foxo1* were generated as described earlier (Kikuchi et al. 2014).

### In vivo studies with entinostat

Orthotopic allograft mouse models of rhabdomyosarcoma were generated as described earlier (Aslam et al. 2014). Once the tumors reached 0.25 cm^3^, the mice were treated with either entinostat (LC Laboratories) at a daily dose of 10 mg/kg by intraperitoneal injection, actinomycin D (Sigma-Aldrich) at 0.25 mg/kg on day 1 by intraperitoneal injection, a combination of entinostat and actinomycin D, or vehicle (DMSO). Once the tumors reached 1.5 cm^3^ or the mice became sick (10%–15% loss of body weight) due to drug toxicity, the mice were euthanized. After 5 d of treatment with entinostat, the mice showed signs of drug toxicity (loss of body weight), so the treatment was halted for a day and then continued with 5 mg/kg daily dose (half the initial dose). All of the drug studies in mice were performed after receiving approval from the IACUC at Oregon Health and Science University.

### Statistical analysis for in vivo studies with entinostat

A repeated measures linear mixed effects model with a compound symmetric autocorrelation assumption to contrast treatments pairwise on day 7 with regard to tumor size was used. Day 7 was chosen for analysis because this was the last day with nearly complete data (actinomycin D, *n* = 7; actinomycin D + entinostat, *n* = 8; DMSO, *n* = 7; entinostat, *n* = 9). The tumor size data were log transformed prior to analysis and corrected the *P*-values for multiple comparisons using the Tukey method.
